# LncRNA-H19 promotes hepatic lipogenesis by directly regulating miR-130a/PPARγ axis in non-alcoholic fatty liver disease

**DOI:** 10.1042/BSR20181722

**Published:** 2019-07-16

**Authors:** Jun Liu, Tao Tang, Guo-Dong Wang, Bo Liu

**Affiliations:** 1 Department of Geriatrics, Xiangya Hospital, Central South University, Changsha 410008, P.R. China; 2 Anhui Provincial Engineering Research Center for Polysaccharide Drugs, School of Pharmacy, Wannan Medical College, Wuhu 241002, P.R. China

**Keywords:** lncRNA-H19, miR-130a, NAFLD, PPARγ

## Abstract

**Background:** As one of the most common liver disorders worldwide, non-alcoholic fatty liver disease (NAFLD) begins with the abnormal accumulation of triglyceride (TG) in the liver. Long non-coding RNA-H19 was reported to modulate hepatic metabolic homeostasis in NAFLD. However, its molecular mechanism of NAFLD was not fully clear.

**Methods:** *In vitro* and *in vivo* models of NAFLD were established by free fatty acid (FFA) treatment of hepatocytes and high-fat feeding mice, respectively. Hematoxylin and Eosin (H&E) and Oil-Red O staining detected liver tissue morphology and lipid accumulation. Immunohistochemistry (IHC) staining examined peroxisome proliferator-activated receptor γ (PPARγ) level in liver tissues. ELISA assay assessed TG secretion. Luciferase assay and RNA pull down were used to validate regulatory mechanism among H19, miR-130a and PPARγ. The gene expression in hepatocytes and liver tissues was detected by quantitative real-time PCR (qRT-PCR) and Western blotting.

**Results:** H19 and PPARγ were up-regulated, while miR-130a was down-regulated in NAFLD mouse and cellular model. H&E and Oil-Red O staining indicated an increased lipid accumulation. Knockdown of H19 inhibited steatosis and TG secretion in FFA-induced hepatocytes. H19 could bind to miR-130a, and miR-130a could directly inhibit PPARγ expression. Meanwhile, miR-130a inhibited lipid accumulation by down-regulating NAFLD-related genes PPARγ, SREBP1, SCD1, ACC1 and FASN. Overexpression of miR-130a and PPARγ antagonist GW9662 inhibited lipogenesis and TG secretion, and PPARγ agonist GW1929 reversed this change induced by miR-130a up-regulation.

**Conclusion:** Knockdown of H19 alleviated hepatic lipogenesis via directly regulating miR-130a/PPARγ axis, which is a novel mechanistic role of H19 in the regulation of NAFLD.

## Introduction

Non-alcoholic fatty liver disease (NAFLD) is one of the most common liver disorders worldwide, which is often related with an unhealthy diet, type 2 diabetes and obesity [1,2]. NAFLD begins with the abnormal accumulation of triglyceride (TG) in the liver and can develop to cirrhosis and even hepatocellular carcinoma (HCC) [2,3]. It has been shown that NAFLD is responsible for over 13% of HCC patients, and the incidence of NAFLD was increasing year by year [4,5]. Therefore, exploring the underlying mechanisms of NAFLD and finding key therapeutic targets are important for the treatment and drug development of NAFLD.

Some important factors control fatty acid synthesis in the liver and play vital role in the process of NAFLD. Sterol-regulatory element binding protein-1 (SREBP-1), a transcription factor of basic helix-loop-helix-leucine zipper family, can transcriptionally activate genes required for lipogenesis such as acetyl-CoA carboxylase (ACC), fatty acid synthetase (FAS) and stearoyl-CoA desaturase (SCD) [6]. Meanwhile, peroxisome proliferator-activated receptor γ (PPARγ) is another major transcription factors and an important member of nuclear receptor superfamily [7]. PPARγ is highly expressed in adipose tissue, and plays an important role in the regulation of lipid metabolism. It was reported that up-regulated PPARγ expression was observed in NAFLD patients, and increased PPARγ activity in liver can lead to storage of lipid in liver [8]. However, the molecular pathogenesis of NAFLD still need further elucidation, and the upstream regulation mechanism of PPARγ in NAFLD is still unclear.

Long non-coding RNAs (lncRNAs) are RNAs longer than 200 nucleotides with limited coding potential, which have many functions and regulate different processes by various molecular mechanisms [9]. H19 was the first lncRNA ever discovered, and has many diverse biological functions, participating in the regulation of cell proliferation, differentiation and metabolism [10]. It was also reported that H19 was abundant in the liver, and played regulatory role in liver development through epigenetic mechanisms [11]. The expression of H19 was increased by fatty acids in hepatocytes and in diet-induced fatty liver, and overexpression of H19 could promote steatosis and augment lipid accumulation [12]. Hepatic overexpression of H19 facilitated the development of obstructive cholestatic liver fibrosis [13]. These implied that H19 may serve as a lipid sensor to modulate hepatic metabolic homeostasis and play a key role in NAFLD, but its underlying molecular mechanism in NAFLD is still unknown.

MicroRNAs (miRNAs) are about 22-nt endogenous RNAs involved in regulating multiple physiological biological processes [14,15]. Increasing evidence from recent studies suggested that miRNAs could regulate a lot of signaling pathways including adipogenesis and lipid metabolism [16]. miR-130a, a member of miR-130 family, was reported to play pivotal roles in fine-tuning of metabolic processes in hepatocytes [17]. Recently, it was reported that miR-130a expression was significantly reduced in livers of NAFLD mouse model and NAFLD patients, and miR-130a could attenuate activation and induce apoptosis of hepatic stellate cells in non-alcoholic fibrosis steatohepatitis [18]. Overexpression of miR-130a contributed to metabolic homeostasis by reducing PPARγ [19]. Additionally, previous study demonstrated that H19 could promote cell migration and invasion by directly targeting miR-130a in glioma [20]. We found that there was a binding site for miR-130a on H19 by bioinformatics analysis. However, whether H19 promotes steatosis through regulating miR-130a in NAFLD has not been reported, which requires further verification

In the present study, we performed a systemic study to investigate the functional implication of H19 in lipid metabolism using both NAFLD mouse and cellular model. Our findings suggested that knockdown of H19 inhibited PPARγ expression to alleviate lipid deposition via directly up-regulating miR-130a. We are the first to reveal that H19 regulates the expression of downstream genes of miR-130a through a ceRNA mechanism and exerts its biological functions in NAFLD. Besides, our data provide a novel perspective showing the regulatory role of H19 in liver tissues, and H19 may be potential target for the treatment of NAFLD.

## Materials and methods

### Mouse model of NAFLD

A total of 12 C57BL/6 mice (males, 8 weeks old) were purchased from SJA Laboratory Animal Corp (China). Mice were divided into two groups (*n*=6 each) and were fed with high-fat diet (HFD) or standard chow diet (control group) for 8 weeks to establish mouse NFALD model. All animal experimental procedures followed the guideline stipulated by Research Ethical Committee for Animal Study.

### Cell culture and treatment

HepG2 and Huh-7 cells were obtained from the American Type Culture Collection (ATCC, U.S.A.) and cultured in Dulbecco’s modified Eagle’s medium (DMEM) (ThermoFisher Scientific, U.S.A.) supplemented with 10% fetal bovine serum (Thermo Fisher Scientific, U.S.A.) at 37°C and 5% CO_2_. For NAFLD cellular model, HepG2 and Huh-7 cells were assigned into control and free fatty acid (FFA; mixture of 1 mM FFAs, containing oleic acid and palmitic acid at 2:1 volume ratio) treatment group to induce steatosis of different degree, and were used for further assays. In the latter group, cells were treated with concentrations of 1 mM FFA for 24 h, and were used for further assays. The PPARγ agonist GW1929 and the PPARγ antagonist GW9662 were purchased from Sigma–Aldrich. HepG2 and Huh-7 cells were treated with 20 μM of GW1929 and 10 μM of GW9662 for 1 h, followed by FFA treatment.

### Cell transfection

The human H19 shRNA vector was purchased for Genechem (Shanghai, China). In brief, H19 shRNA sequence was synthesized and subcloned into transfection plasmid to generate recombinant vector. Then, there combinant vector was transfected into HepG2 and Huh-7 cells with Lipofectamine® 2000 Reagent (Invitrogen, U.S.A.) following the manual instruction. miR-130a mimics and inhibitor were purchased from GenePharma (Shanghai, China) and were transfected into hepatocytes by lipofectamine RNAiMAX (Invitrogen, U.S.A.) according to the manufacturer’s instructions.

### ELISA assay

Extraction and measurement of TG in liver tissues or cells were performed as described before [21]. Briefly, the supernatant of cultured medium was extracted for quantifying the concentration of TG using ELISA kits (Applygen, China) following manual instructions. For NAFLD mice, liver tissues were collected after sacrifice, and were homogenized to extract the supernatant. TG levels were then determined using ELISA kits.

### Luciferase activity assay

Luciferase reporter assay was performed by co-transfecting firefly luciferase reporter plasmid containing H19 wild-type or mutant (pGL3-H19 WT/MUT-firefly luciferase), or 3′-UTR wild-type or mutant of PPARγ mRNA (pGL3-PPARγ WT/MUT-firefly luciferase), *Renilla* luciferase control reporter vector (pRL, Promega, U.S.A.), and miR-130a inhibitor or miR-130a mimics or scramble RNAs into HepG2 and Huh-7 cells by Lipofectamine® 2000 Reagent (Invitrogen, U.S.A.). Dual-Luciferase Reporter Assay System (Promega, U.S.A.) was used after transfection to examine the relative luciferase activity.

### Pull-down assay

Pull-down assay was carried out as described before [22]. Briefly, biotinylated miR-130a, miR-130a-Mut and negative control of miR-130a were purchased from GenePharma (Shanghai, China) and were transfected into HepG2 and Huh-7 cells. Cells were harvested after transfection for 48 h. A total of 50 μl of samples were then aliquot for input. Dynabeads M-280 Streptavidin (Invitrogen, U.S.A.) was then used to incubate the remaining samples according to the manufacturer’s protocol. Beads were washed and treated in RNase-free solutions, then incubated with equal volumes of biotinylated miR-130a for 15 min at room temperature using gentle rotation. Beads with the immobilized miR-130a fragment were incubated in 10 mmol/l ethylenediaminetetraacetate, pH 8.2 with 95% formamide for 5 min at 65°C. RNAs were purified and assessed by quantitative real-time PCR (qRT-PCR).

### Hematoxylin–Eosin (H&E) staining

Hematoxylin and Eosin (H&E) staining was performed as described before [23]. Briefly, tissues were fixed in 4% formaldehyde in PBS, and then stained with H&E. Then a standard microscopy (Olympus Imaging America Inc., U.S.A.) was used to acquire images.

### Oil-Red O staining

Frozen sections of liver specimens and cells were fixed in 4% paraformaldehyde for 10 min after certain treatment and washed three times with PBS buffer. Then, samples were incubated with Oil-Red O staining solution for 10 min and washed with 60% isopropanol once and then PBS twice. For nuclear staining, hematoxylin was added and washed with PBS for three times. Then, a light microscopy (Nikon, Japan) was used to inspect samples.

### Immunohistochemistry

Immunohistochemistry (IHC) were performed as previously described [24]. Briefly, liver tissues were fixed in 4% formalin for 24 h, and then embedded in paraffin. Then, IHC was carried out with MaxVision™ techniques (Maixin-Bio, China) according to the manufacturer’s instructions. After blocking the endogenous peroxides and proteins, it was incubated with primary antibodies specific for PPARγ (abcam, U.S.A., 1:200) overnight at 4°C. Then, the slices were stained with a 3,3′-diaminobenzidine solution for 3 min and counterstained with haematoxylin.

### RNA extraction and quantitative real-time PCR

Total RNA of cells and liver tissues was isolated using Trizol (Invitrogen, U.S.A.). ImProm-II Reverse Transcription System (Promega, U.S.A.) was used to generate First-strand cDNA. SYBR Green qPCR assay (Takara, China) and gene-specific primers were used for qRT-PCR with β-actin or U6 used for normalization following the manufacturer’s protocol. The following conditions were used to carry out qRT-PCR: 95°C for 10 min, followed by 40 cycles each containing 95°C for 15 s, 60°C for 20 s and 72°C for 40 s. The relative expression levels of RNAs were calculated using the comparative Ct method. Each sample was tested in triplicates for statistical analysis. The primers used in the present study are provided in Table 1.

**Table 1:** Primers used for qRT-PCR analysis

Genes	Primer sequences (5′-3′)
*has(mmu)-miR-130a*	F: 5′-GCCGCAGTGCAATGTTAAAA-3′
	R: 5′-GTCGTATCCAGTGCAGGGTCCGAGGT
	ATTCGCACTGGATACGACATGCCC-3′
*has(mmu)-U6*	F: 5′-CTCGCTTCGGCAGCACA-3′
	R: 5′-AACGCTTCACGAATTTGCGT-3′
*hH19*	F: 5′-TCTGGCAGGAGTGATGACGG-3′
	R: 5′-CAGGAGAGTTAGCAAAGGTG-3′
*hPPARγ*	F: 5′-CTCAAACGAGAGTCAGCCTT-3′
	R: 5′-GAGTGGGAGTGGTCTTCCAT-3′
*hSREBP1*	F: 5′-GGAGCCATGGATTGCACTTTCG-3′
	R: 5′-GCTCAGGAAGGCTTCAAGAGAG-3′
*hFASN*	F: 5′-TATGAAGCCATCGTGGACGG-3′
	R: 5′-GAAGAAGGAGAGCCGGTTGG-3′
*hSCD1*	F: 5′-CTTGCGATATGCTGTGGTGC-3′
	R: 5′-AAGTTGATGTGCCAGCGGTA-3′
*hACC1*	F: 5′-CAAGGTCAGCTGGTCCACATG-3′
	R: 5′-GTGGAATACCTTCTGCCCTAGC-3′
*hβ-actin*	F: 5′-CCTCGCCTTTGCCGATCC-3′
	R: 5′-GGATCTTCATGAGGTAGTCAGTC-3′
*mH19*	F: 5′-AAGAGCTCGGACTGGAGACT-3′
	R: 5′-AAGAAGGCTGGATGACTGCC-3′
*mPPARγ*	F: 5′-AAGAGCTGACCCAATGGTTG-3′
	R: 5′-ACCCTTGCATCCTTCACAAG-3′
*mSREBP1*	F: 5′-CCACAATGCCATTGAGAAGCG-3′
	R: 5′-CTGACACCAGGTCCTTCAGTG-3′
*mFASN*	F: 5′-TTGCTGGCACTACAGAATGC-3′
	R: 5′-AACAGCCTCAGAGCGACAAT-3′
*mSCD1*	F: 5′-CACACCTTCCCCTTCGACTA-3′
	R: 5′-TGACTCCCGTCTCCAGTTCT-3′
*mACC1*	F: 5′-CTTGGAGCAGAGAACCTTCG-3′
	R: 5′-ACTTCCCGACCAAGGACTTT-3′
*mβ-actin*	F: 5′-GTCGTACCACAGGCATTGTGATGG-3′
	R: 5′-GCAATGCCTGGGTACATGGTGG-3′

### Western blotting

Liver tissues and cells were harvested and isolated the protein with RIPA lysis buffer (Thermo Fisher Scientific, U.S.A.) containing protease inhibitors (Roche, Switzerland) and phosphatase inhibitors (Sigma–Aldrich, U.S.A.). After BCA kit (Pierce, U.S.A.) quantification, equal amounts of protein samples were separated by 10% SDS-PAGE and then transfer to PVDF membranes. The membrane was incubated with a specific primary antibody PPARγ (1:1000, abcam, U.S.A.), ACC1 (1:1000, abcam, U.S.A.), SCD1 (1:1000, abcam, U.S.A.), FASN (1:1000, abcam, U.S.A.) and SREBP1 (1:1000, abcam, U.S.A.). Quantitative autoradiography was performed by optical density method using GAPDH (1:5000, proteintech, U.S.A.) as controls, followed by appropriate secondary antibodies (1:5000, Pierce, U.S.A.). Immobilon Western chemiluminescent HRP substrate (Millipore, U.S.A.) was used to visualize signals and film exposure.

### Statistical analysis

GraphPad Prism 7 software was used to calculate and assess statistical differences between experimental groups. The results were presented as mean ± Standard Deviation (SD). Comparison between two groups was performed using two-tailed Student’s *t* test and for multi-group comparison one-way ANOVA test was used. *P*<0.05 was considered statistically significant (*P<0.05 and **P<0.01).

## Results

### H19 is up-regulated and hepatic lipogenesisis is increased in NAFLD mouse model

**Figure 1:**
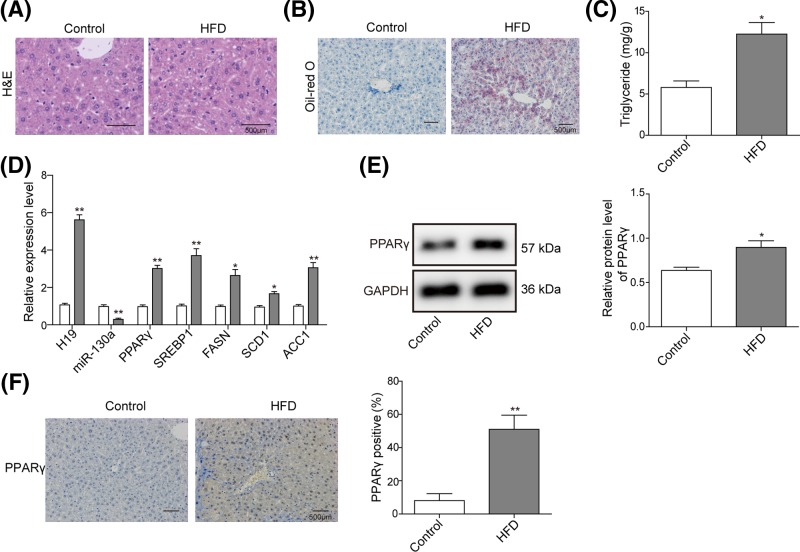
The expression level of H19 and hepatic lipogenesisis in NAFLD mouse model (**A**) H&E staining showed general tissue morphology of mouse livers between control and HFD groups. (**B**) Oil-Red O staining showed prominent lipid deposition in HFD mice. (**C**) TG levels were assessed by ELISA assay in liver tissues. (**D**) qRT-PCR was used to detect the level of H19, miR-130a and molecular marker of NAFLD (PPARγ, ACC1, SCD1, FASN and SREBP1) in NAFLD mouse liver tissues. β-actin or U6 were used for normalization. (**E**) Western blot analysis of PPARγ level in NAFLD mouse liver tissues. GAPDH was used for normalization. (**F**) IHC detection of PPARγ in NAFLD mouse liver tissues. All the results were shown as mean ± SD (*n*=3), which were three separate experiments performed in triplicate. *P<0.05 and **P<0.01.

To identify the effect of lipid accumulation on the expression of H19, NAFLD mice model was conducted by HFD feeding. H&E staining of liver tissues showed enlarged hepatocyte volume and dispersion of lipid vacuoles in model group compared with control group (Figure 1A), and Oil-Red O staining showed remarkable deposition of lipid droplet with liver (Figure 1B), indicating an increased hepatic lipid accumulation. Consistently, TG level was significantly increased in HFD group compared with control group (Figure 1C). Further, qRT-PCR assay revealed that H19 as well as NAFLD-related genes (PPARγ, ACC1, SCD1, FASN and SREBP1) were significantly increased in the liver of HFD mice compared with control group (Figure 1D), while miR-130a was down-regulated in the liver of HFD mice. Moreover, as PPARγ plays an important role in the transcriptional regulation of lipid metabolism, we detected PPARγ protein level in NAFLD mouse model. Western blotting revealed that PPARγ protein was significantly increased in NAFLD mouse model (Figure 1E). While more PPARγ-positive cells were detected in NAFLD group by IHC assays (Figure 1F). These results indicated that H19 and PPARγ were up-regulated in NAFLD mouse model.

### FFA promotes H19 expression and facilitates lipogenesis in hepatocytes

**Figure 2:**
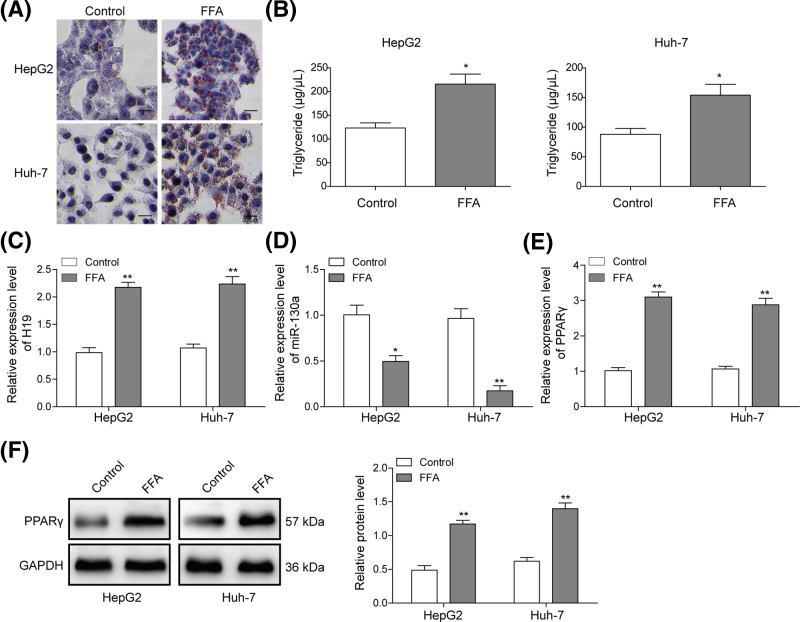
FFA induced hepatocyte lipid degeneration and increased H19 expression in hepatocytes (**A**) Oil-Red O staining for lipid droplet in HepG2 and Huh-7 cells treated with FFA. (**B**) TG levels were detected by ELISA assay in HepG2 or Huh-7 cells treated with FFA. Relative expression level of H19 (**C**), miR-130a (**D**) and PPARγ (**E**) was detected by qRT-PCR in HepG2 or Huh-7 cells treated with FFA. (**F**) Protein level of PPARγ was evaluated by Western blotting in HepG2 or Huh-7 cells treated with FFA. GAPDH was used for normalization. All the results were shown as mean ± SD (*n*=3), which were three separate experiments performed in triplicate. *P<0.05 and **P< 0.01.

Next, we used NAFLD cellular model to evaluate the expression and role of H19. As shown in Figure 2A, both in FFA-treated HepG2 and Huh-7 cells, the accumulated lipid increased significantly, which was examined by Oil-Red O staining. Consistently, TG level was significantly increased in HepG2 and Huh-7 cells treated with FFA (Figure 2B). Then, the expression of H19, miR-130a and PPARγ was detected by qRT-PCR. As shown in Figure 2C–E, H19 and PPARγ were up-regulated in NAFLD cellular model, while miR-130a was decreased significantly. Furthermore, the protein level of PPARγ was also detected by Western blotting. PPARγ protein was also up-regulated in FFA-treated HepG2 and Huh-7 cells (Figure 2F). These results demonstrated that H19 may up-regulate the expression of lipogenesis genes for increasing lipid deposition or secretion from hepatocytes induced by FFA.

### H19 directly down-regulates the expression of miR-130a

**Figure 3:**
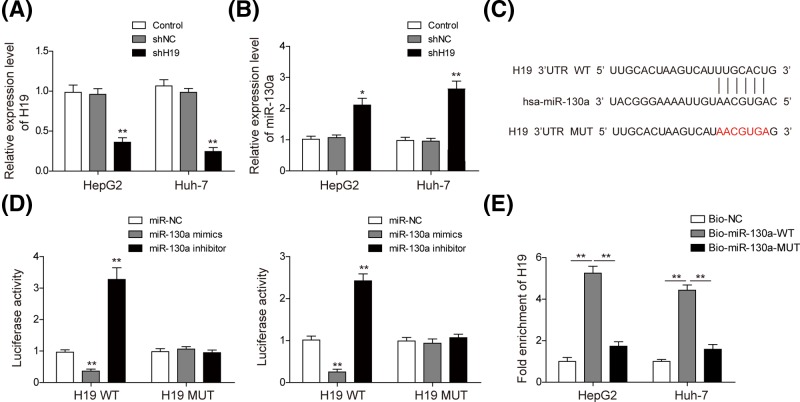
H19 could directly regulate expression of miR-130a Relative expression level of H19 (**A**) and miR-130a (**B**) was measured by qRT-PCR in HepG2 and Huh-7 cells transfected with shH19. (**C**) Predicted binding site between miR-130a and H19 by TargetScan software. (**D**) The luciferase activity of the H19-WT and H19-MUT in HepG2 and Huh-7 cells transfected with miR-130a mimics and inhibitor. (**E**) PCR was used to detect H19 in the samples pulled down by biotinylated miR-130a. All the results were shown as mean ± SD (*n*=3), which were three separate experiments performed in triplicate. *P<0.05 and **P<0.01.

To investigate the regulatory role of H19, we conducted H19 knockdown with H19 shRNA in HepG2 and Huh-7 cells. As shown in Figure 3A, H19 expression was significantly down-regulated in both cell lines transfected with shH19, indicating that H19 was successfully knockdown. Then, miR-130a expression was also detected after H19 knockdown. Figure 3B showed that miR-130a was increased in H19 knockdown cells. We further predicted the potential binding site between H19 and miR-130a through bioinformatics analysis (Figure 3C). Then, to determine whether H19 directly regulates miR-130a, luciferase reporter vectors of wild-type H19 (H19-WT) and a mutant form (H19-MUT) were constructed to carry out dual luciferase reporter assay. Dual luciferase reporter assay confirmed that overexpression of miR-130a suppressed the luciferase activity of the H19-WT in HepG2 and Huh-7 cells significantly, with no effect observed on the mutant form (Figure 3D). These results indicated direct interaction of H19 with miR-130a in this putative binding site, and H19 could negatively regulate the expression of miR-130a. Moreover, we used a biotin pull-down system to investigate whether miR-130a could pull down H19. As shown in Figure 3E, H19 was pulled down by miR-130a, but the miR-130a mutation group was unable to pull down H19, meaning that recognition between H19 and miR-130a was specific.

### Knockdown of H19 inhibits FFA-induced lipid accumulation and TG secretion in hepatocytes

**Figure 4:**
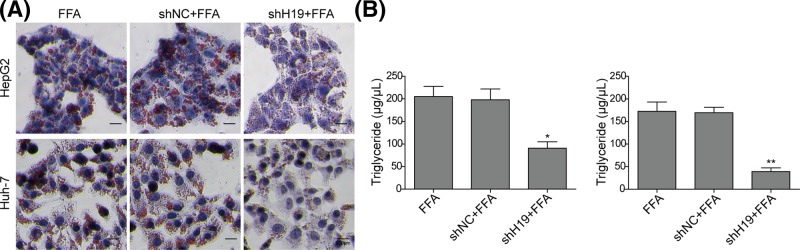
Knockdown of H19 inhibited steatosis and TG secretion in FFA-induced hepatocytes (**A**) Oil-Red O staining for lipid droplet in FFA-challenged HepG2 and Huh-7 cells transfected with shH19. (**B**) TG levels were detected by ELISA assay in FFA-challenged HepG2 and Huh-7 cells transfected with shH19. All the results were shown as mean ± SD (*n*=3), which were three separate experiments performed in triplicate. *P<0.05 and **P<0.01.

To investigate whether H19 could regulate hepatocyte steatosis, we examined the effects of H19 on lipid accumulation and TG secretion in both cell lines. The Oil-Red O staining was used to examine lipid accumulation. As shown in Figure 4A, the lipid droplets accumulation did not show much difference between FFA-treated group and sh-NC-transfected FFA-treated group, while the lipid droplets accumulation was significantly reduced in both HepG2 and Huh-7 cells treated with shH19 compared with NC groups, indicating that knockdown of H19 could inhibit hepatocyte steatosis. Consistently, significant decreases in TG were observed in cells treated with shH19 (Figure 4B). These results fully indicated that H19 knockdown inhibits FFA-induced lipid accumulation and TG secretion in hepatocytes.

### miR-130a inhibits PPARγ expression via binding to 3′-UTR of PPARγ

**Figure 5:**
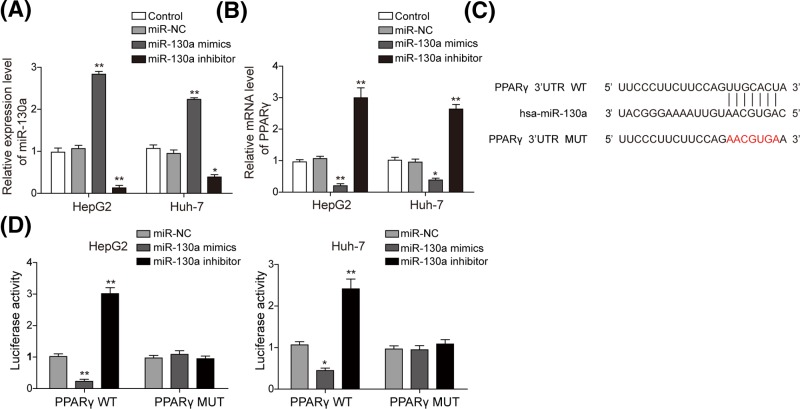
miR-130a inhibited the expression of PPARγ by binding with its 3′-UTR Relative expression level of miR-130a (**A**) and PPARγ (**B**) was measured by qRT-PCR in HepG2 and Huh-7 cells transfected with miR-130a mimics and inhibitor. (**C**) Predicted binding site between miR-130a and PPARγ by TargetScan software. (**D**) The luciferase activity of the PPARγ-WT and PPARγ-MUT in HepG2 and Huh-7 cells transfected with miR-130a mimics and inhibitor. All the results were shown as mean ± SD (*n*=3), which were three separate experiments performed in triplicate. *P<0.05 and **P<0.01.

It was reported that PPARγ was the target of miR-130a in bovine mammary epithelial cells [19], but it is unknown whether miR-130a plays a role in NAFLD by targeting PPARγ. To investigate whether miR-130 could bind and regulate the expression of PPARγ in hepatocytes, miR-130a mimics and miR-130a inhibitor were transfected to both cell lines. Figure 5A indicated that miR-130a was significantly increased by miR-130a mimics while it was significantly down-regulated by its inhibitor. PPARγ was significantly increased in miR-130a inhibitor group while was significantly decreased in miR-130a mimics group (Figure 5B). Bioinformatics analysis found the potential binding site between miR-130a and PPARγ (Figure 5C). Further dual luciferase reporter assay confirmed that miR-130a could directly target on 3′-UTR of PPARγ (Figure 5D). When treated with miR-130a mimics/inhibitor, the relative luciferase activity significantly decreased/increased in PPARγ-WT but did not change in the PPARγ-MUT (Figure 5D). These results suggested that miR-130a directly and negatively regulates the expression of PPARγ through binding the 3′-UTR region of PPARγ.

### miR-130a regulates the hepatocyte steatosis via directly regulating PPARγ signal

**Figure 6:**
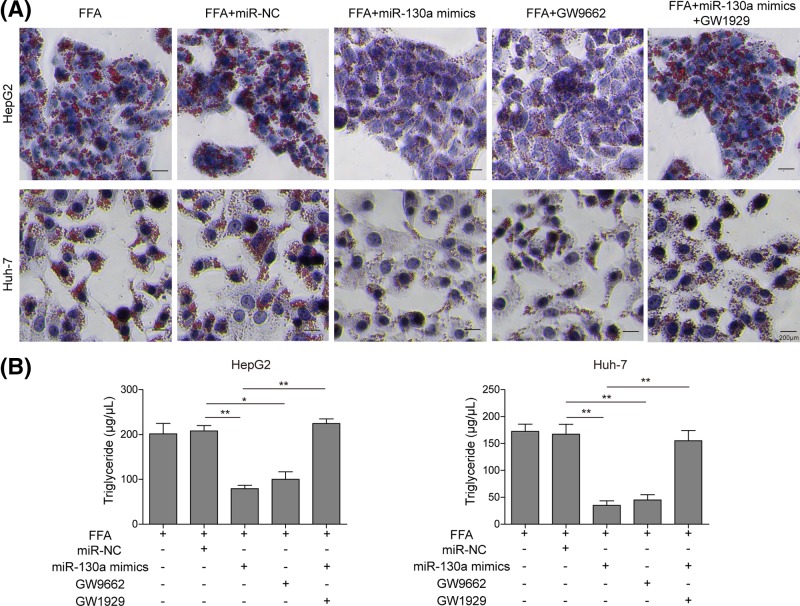
Overexpression of miR-130a inhibited steatosis and TG secretion by directly targeting PPARγ in FFA-induced hepatocytes (**A**) Oil-Red O staining for lipid droplet in FFA-challenged HepG2 and Huh-7 cells treated with miR-NC, miR-130a mimics, GW9662 and miR-130a mimics with GW1929. (**B**) TG levels were detected by ELISA assay in FFA-challenged HepG2 and Huh-7 cells treated with miR-NC, miR-130a mimics, GW9662 and miR-130a mimics with GW1929. All the results were shown as mean ± SD (*n*=3), which were three separate experiments performed in triplicate. *P<0.05 and **P<0.01.

To confirm miR-130a could regulate the hepatocyte steatosis through PPARγ, we examined the effects of PPARγ on lipid accumulation and TG secretion in HepG2 and Huh-7 cells treated with PPARγ agonist (GW1929), antagonist (GW9662) or miR-130a mimics. As shown in Figure 6A, GW9662 administration markedly reduced lipid droplets accumulation in both HepG2 and Huh-7 cells, indicating that PPARγ could facilitate hepatocyte steatosis. Furthermore, miR-130a mimics reduced lipid droplets accumulation in both HepG2 and Huh-7 cells, while GW1929 treatment could compensate this effect, indicating that miR-130a could regulate the hepatocyte steatosis through PPARγ. Consistently, significant decrease in TG was observed in cells treated with GW9662 (Figure 6B). miR-130a mimics reduced TG, while GW9662 treatment could reverse this effect (Figure 6B). These results fully indicated that PPARγ facilitates FFA-induced lipid accumulation and TG secretion in hepatocytes, through which miR-130a could inhibit hepatocyte steatosis.

### miR-130a inhibits hepatocyte steatosis via modulating lipogenesis-related genes expression

**Figure 7:**
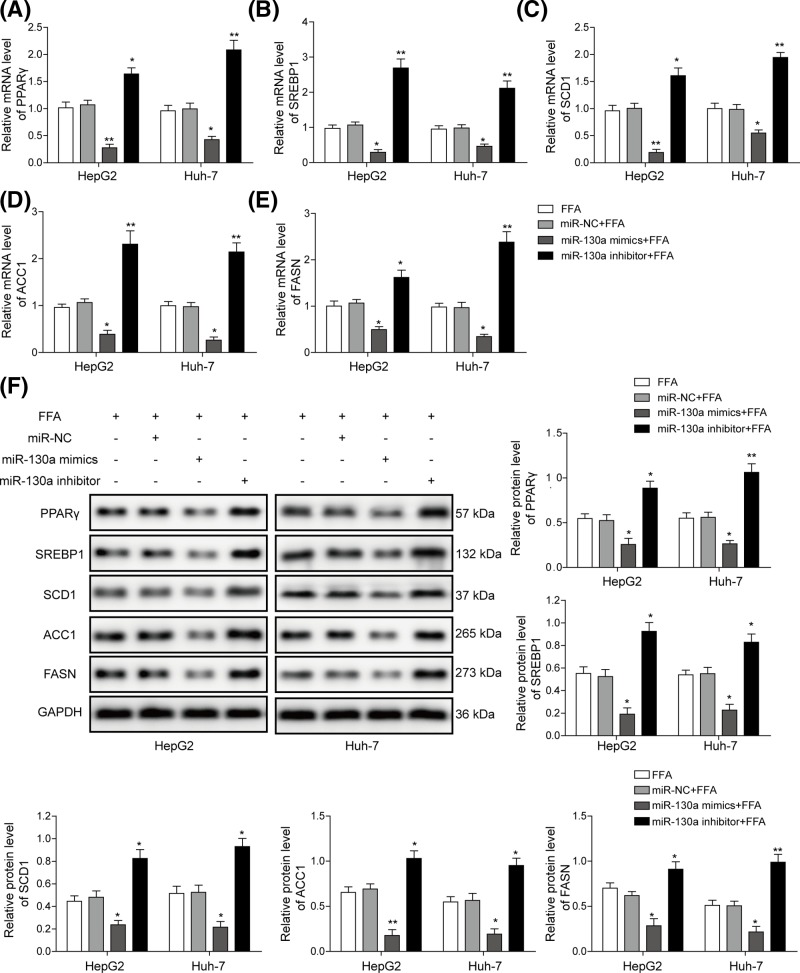
miR-130a inhibited expression of NAFLD-related genes Relative expression level of PPARγ (**A**), SREBP1 (**B**), SCD1 (**C**), ACC1 (**D**) and FASN (**E**) was measured by qRT-PCR in FFA-challenged HepG2 and Huh-7 cells transfected with miR-130a mimics and inhibitor. (**F**) Relative protein level of lipogenesis-related genes was evaluated by Western blotting in FFA-challenged HepG2 and Huh-7 cells transfected with miR-130a mimics and inhibitor, GAPDH was used for normalization. All the results were shown as mean ± SD (*n*=3), which were three separate experiments performed in triplicate. *P<0.05 and **P<0.01.

To explore the effect of miR-130a on hepatocyte steatosis, we detected lipogenesis-related genes in FFA-induced HepG2 and Huh-7 cells treated with miR-130a mimics and miR-130a inhibitor. Lipid metabolism is regulated by various genes including PPARγ, SREBP1, SCD1, ACC1 and FASN. qRT-PCR assay suggested that FFA treatment significantly elevated expression level of all those genes (Figure 7A–E). All the five genes were down-regulated in FFA-induced HepG2 and Huh-7 cells treated with miR-130a mimics, while significantly up-regulated in miR-130a inhibitor group compared with FFA group (Figure 7A–E). The expression of these proteins by Western blotting uncovered that these proteins were down-regulated in HepG2 and Huh-7 cells transfected with miR-130a mimics, while significantly up-regulated in miR-130a inhibitor group compared with NC group (Figure 7F). Therefore, miR-130a could repress lipogenesis-related genes, thus alleviating FFA-induced lipid droplet formation and secretion.

## Discussion

NAFLD now has a prevalence ∼24% worldwide [4], and excessive accumulation of TGs in the hepatocytes without consumption of alcohol is its main characteristic [6]. Previous studies showed that lncRNA had a variety of biological functions and was involved in the regulation of the development of various diseases, but there were few studies on lncRNA in NAFLD. In the present study, we for the first time demonstrated that H19 could promote hepatic lipogenesis via miR-130a/PPARγ axis. The present study also provided a mechanistic role for lncRNAs in regulating the development of NAFLD, which could be a potential treatment target for NAFLD.

Previous study showed that H19 could participate in the regulation of various biological processes such as cell proliferation, apoptosis and metabolism [25], and H19 was up-regulated in cirrhotic liver tissue [26]. H19 was also reported to be a lipid sensor by synergizing with polypyrimidine tract-binding protein 1 (PTBP1) to modulate hepatic metabolic homeostasis [12]. However, the underlying molecular mechanism of H19 in NAFLD is still unclear. In the present study, H19 was observed to be up-regulated in NAFLD animal model and cellular model and knockdown H19 can inhibit lipid accumulation and TG secretion in FFA-induced HCC cells, which is consistent with previous research, indicating that H19 plays regulatory roles in NAFLD progression.

Recent many studies suggested that lncRNAs functions as miRNA sponges to modulate the derepression of miRNA targets [27]. H19 regulated EZH2 and promoted cell invasion by interacting with miR-630 in nasopharyngeal carcinoma [28]. Furthermore, H19 could regulate laryngeal squamous cell cancer progression by miR-148a-3p/DNMT1 axis [29]. H19 was closely related to liver diseases and was up-regulated in human chronic liver diseases [30]. H19 was up-regulated in NAFLD cell and mouse model, and overexpression of H19 could promote steatosis and lipid accumulation [12]. Recently, miR-130a, a well-documented miRNA, was predicted to be involved in NAFLD [31]. miR-130a was significantly decreased by miRNA microarray analysis in liver tissue of NAFLD mice [32], and miR-130a could inhibit adipogenesis in human primary preadipocytes [33]. Besides, miR-130a was reported to target PPARγ [34]. However, the relationship between H19 and miR-130a in NAFLD has not been reported yet. Inspired by these studies, we proposed that H19 could play a regulatory role in the progression of NAFLD by sponging miR-130a. We uncovered that miR-130a was down-regulated in NAFLD mouse model and FFA-induced hepatocytes, and miR-130a expression was up-regulated after H19 knockdown. Moreover, the expression of PPARγ was negatively correlated with miR-130a. We further validated that miR-130a could directly bind with H19 and PPARγ through bioinformatics analysis and luciferase assays. Taken together, these data indicated that H19 may regulate PPARγ expression through targeting miR-130a.

PPAR is one of the important regulators and are associated with nutrient metabolism [7]. PPARγ, one of its subtype, was reported to play an key role in the transcription process, glucose metabolisms and other events, especially an indispensable role in adipogenesis, which was frequently impaired under pathological conditions such as NAFLD or non-alcoholic steatohepatitis (NASH) [21,35]. PPARγ as well as SREBP1, SCD1, ACC1 and FASN are transcription factors that control genes involved in fatty acid and TG synthesis [36,37]. Among them, SCD1 and ACC1 are SREBP1 target genes transcriptionally regulated by SREBP1 involving in the pathway of modification of FA chain or FA storage [38,39]. In the present study, we uncovered that PPARγ protein was significantly increased in NAFLD mouse model and FFA-treated cell model. PPARγ facilitated FFA-induced lipid accumulation and TG secretion in hepatocytes. miR-130a regulated lipogenesis in hepatocytes through targeting PPARγ.

In summary, we demonstrated that elevated H19 expression is a characteristic molecular change in NAFLD, and H19 promotes hepatocyte steatosis, TG secretion and expression of NAFLD-related genes via activating PPARγ signal by sponging miR-130a. Taken together, our study confirms a regulatory role of lncRNAs in regulating NAFLD progression, and H19 may be a key and novel molecular target in NAFLD.

## Abbreviations

ACC: acetyl-CoA carboxylase
FASN: fatty acid synthase
FFA: free fatty acid
GAPDH: glyceraldehyde-3-phosphate dehydrogenase
HCC: hepatocellular carcinoma
HFD: high-fat diet
IHC: Immunohistochemistry
lncRNA: long non-coding RNA
miRNA: microRNA
NAFLD: non-alcoholic fatty liver disease
NC: negative control
PPARγ: peroxisome proliferator-activated receptor γ
PVDF: polyvinylidene fluoride
qRT-PCR: quantitative real-time PCR
SCD: stearoyl-CoA desaturase
SD: standard deviation
TG: triglyceride
